# Oral Microbiota Associated with Clinical Efficacy of Ustekinumab in Crohn’s Disease

**DOI:** 10.2174/0118715303363951241209060903

**Published:** 2025-01-13

**Authors:** Feiyang Xu, Rui Xie, Le He, Honggang Wang, Yifan Zhu, Xiaozhong Yang, Huiming Yu

**Affiliations:** 1 Department of Stomatology, The Affiliated Huaian No.1 People’s Hospital of Nanjing Medical University, No.1 Huanghe West Road, Huaian, 223300, Jiangsu Province, China;; 2 Department of Gastroenterology, The Affiliated Huaian No.1 People’s Hospital of Nanjing Medical University, No.1 Huanghe West Road, Huaian, 223300, Jiangsu Province, China

**Keywords:** Crohn's disease, ustekinumab, oral microbiota, 16S rRNA, prognostic biomarker, non-invasive prognostic biomarker

## Abstract

**Background:**

Crohn’s Disease (CD) is a chronic inflammatory gastrointestinal disease. Ustekinumab (UST) has been utilized as a therapeutic option for CD patients. However, approximately 40-60% of patients exhibit an inadequate response to UST. Accumulating evidence has confirmed the involvement of oral bacteria in the development of CD. Nevertheless, the relationship between oral microbiota and the efficacy of UST therapy in CD patients has remained unexplored.

**Materials and Methods:**

We recruited 28 healthy individuals and 53 CD patients, 47 of whom completed the entire UST therapy. Oral samples and clinical data were collected. The clinical response and clinical remission were defined based on the CDAI score. Oral samples were analyzed by 16S rRNA gene sequencing. The analysis of sequence data was performed by QIIME and R.

**Results:**

We revealed the oral microbial difference between the Healthy Control (HC) group and the CD group. The enrichment of *Fusobacteria*, *Leptotrichia*, *Capnocytophaga*, and *Campylobacter*, and the diminution of *Haemophilus* and *Rothia* were observed in the CD group. Differences in oral microbiota were also identified among patients with different efficacy of UST. Compared to the response and remission groups, both the non-response and non-remission groups showed significantly higher levels of *Fusobacteria* and *Leptotrichia*. Predictive models for clinical response and clinical remission in UST were developed based on oral microbiota, with the Area Under the Curve (AUC) value of 0.944 and 0.930, respectively.

**Conclusion:**

Oral microbiota was relevant to the UST efficacy in patients with CD based on the predictive model. These findings suggest that oral microbiota could serve as a non-invasive prognostic biomarker for UST treatment in CD patients.

## 1 INTRODUCTION

Crohn’s Disease (CD), a category of Inflammatory Bowel Disease (IBD), can affect any segment of the gastrointestinal tract, from the oral cavity to the anus [1]. The prevalence of CD in North America is reported to be 201 cases per 100,000 individuals, with an annual incidence ranging from 3.1 to 20.2 cases per 100,000. In recent years, the incidence of CD has been rising in Africa, Asia, and South America [2-4]. At diagnosis, most CD patients present with an inflammatory phenotype. However, over time, complications, such as strictures, fistulas, and abscesses, develop in about half of these patients, significantly affecting their quality of life and social relationships [5].

Ustekinumab (UST), a monoclonal antibody, has been used in the treatment of CD patients. It can block the inflammatory pathways by antagonizing the p40 subunit of Interleukin (IL)-12 and IL-23 [6]. However, the clinical efficacy of UST therapy varies among CD patients. According to Feagan, *et al.* [7], about 40% of CD patients lose response to UST. A study by Johnson, *et al.* [8] reported that around 60% of CD patients failed to achieve clinical remission after 12 months of UST therapy. To optimize UST treatment, identifying pre-treatment predictors of efficacy is essential. Factors, such as disease severity, previous surgeries, prior exposure to anti-tumor Necrosis Factor (TNF) therapies, current opioid use, and corticosteroid use have been found to be associated with UST efficacy in CD [9-11]. Besides, biochemical parameters, such as serum Monocyte Chemoattractant Protein-1 (MCP-1) and anti-UST antibodies, have been identified as biological predictors of UST efficacy in CD [12, 13].

Several studies have detected the presence of oral bacteria in the colonic mucosa of CD, especially active CD [14-16]. In pediatric CD, the network of oral bacteria on the colonic mucosa surfaces was found to be similar to that on oral mucosa [17]. Xun, *et al.* [18] observed differences in the oral microbiota between healthy controls and CD patients and, by hierarchical clustering of the oral microbial community at the genus level, identified two distinct microbial ecotypes in CD patients. These findings suggest that oral bacteria may play a role in the development of IBD through ectopic colonization and immune-mediated mechanisms [19-21].

Oral microbiota has also been shown to predict the effectiveness of anti-TNF-α treatment in IBD [22]. Even though the collection of oral samples, when compared to blood and fecal samples, is less invasive and convenient, little is known about the oral microbiota characteristics in CD patients commencing UST. Therefore, our study aimed to explore the relationship between oral microbiota composition and UST treatment outcomes, with the goal of predicting UST efficacy using oral microbiota profiles.

## 2 METHODS

### 2.1 Subjects

**Fig. (1):**
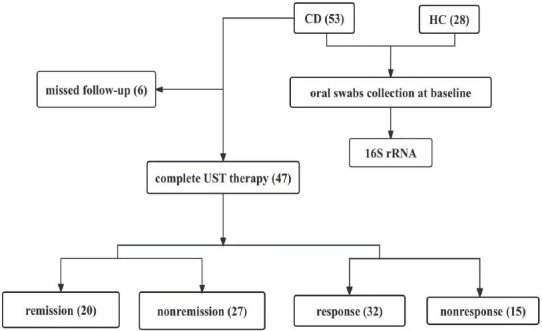
Flow chart (**Abbreviations:** CD: Crohn’s disease group; HC: Healthy control group; UST: Ustekinumab).

Between January 2022 and August 2023, 81 participants, consisting of 28 healthy individuals and 53 patients diagnosed with CD, were recruited for our study at the Affiliated Huaian No.1 People’s Hospital of Nanjing Medical University. Among the CD patients, 47 successfully completed the entire UST therapy (Fig. **1**).

The inclusion criteria for participants diagnosed with Crohn's Disease (CD) were as follows: 1) a confirmed diagnosis of CD for at least three months prior to enrollment, and 2) an initial treatment regimen including inhibitors of Interleukin-12 (IL-12) or Interleukin-23 (IL-23). Exclusion criteria encompassed 1) previous bowel surgery, 2) history of any tumor, 3) presence of periodontitis, 4) oral mucosal diseases or lesions, 5) use of antibiotics or probiotics within the last three months, and 6) current active or opportunistic infections.

Family members of the CD patients, accompanying them to the hospital, were considered for the control group, serving as Healthy Controls (HC). The criteria for inclusion as healthy controls required 1) the absence of gastrointestinal diseases and other systematic diseases, 2) no periodontitis, 3) no oral mucosal diseases or lesions, 4) no history of tumors, 5) no use of antibiotics or probiotics within the preceding three months, and 6) no current active or opportunistic infections.

The demographic data of subjects were collected at the beginning. The schedule of UST therapy in CD patients was as follows: an intravenous injection at baseline (patients weighing less than 55 kg: 260 mg; patients weighing between 55 and 85 kg: 390 mg; patients weighing over 85 kg: 520 mg), following a subcutaneous injection of 90 mg for 8 weeks.

### 2.2 Oral Swab Collection and Oral Examination

Oral swabs were collected from healthy individuals and CD patients at baseline. Following a 12-hour fast, oral swab specimens were gathered between 9 a.m. and 11 a.m. We obtained the oral swabs by gently rubbing both sides of the buccal mucosa surface for 10 seconds. Then all samples were stored at -80°C until DNA extraction. After the collection of oral swabs, an oral lesion examination was performed by visual inspection. The periodontal examination was conducted by measuring Probing Depth (PD) and Clinical Attachment Loss (CAL) at six sites of each tooth.

### 2.3 16S rRNA Sequencing

Genomic DNA from the microbial community was extracted from 81 samples using the E.Z.N.A.^®^Tissue DNA kit (Omega Bio-tek, Norcross, GA, U.S.), following the manufacturer’s instructions. The V3-V4 region of the bacterial 16S rRNA was amplified by forward primer 338F (5’- ACTCCTACGGGAGGCAGCAG-3’) and reverse primer 806R (5’- GGACTACHVGGGTWTCTAAT-3’). We used UNOISE3 to denoise the high-quality sequences, identifying Amplicon Sequence Variants (ASVs). The BLAST tool was used to classify all sequences into different taxonomic groups against the SILVA138 database [23]. The similarity threshold used in BLAST was 0.9. The rarefaction analysis was utilized to assess sequencing depth.

### 2.4 The Assessment of Clinical Outcomes

CD patients who completed the entire UST therapy were categorized into the response group and non-response group, based on the change in Crohn's Disease Activity Index (CDAI) score (Table **S1**). Additionally, the efficacy of UST was assessed using clinical remission as another outcome variable, and then the CD patients were classified into the remission group and the non-remission group. Clinical remission was defined as a CDAI score below 150 points, while clinical response was defined as a decrease in CDAI by a minimum of 100 points compared to the baseline or a CDAI score below 150 points. The evaluations of CDAI score were performed at week 0 and week 16/24.

### 2.5 Oral Microbiota Analysis

Species diversity was analyzed using QIIME (v1.8.0) [24], and R (v3.6.0) was utilized to generate images based on the ASV information. The beta diversity was analyzed by adopting the Principal-coordinate Analysis (PCoA). The distance matrix was calculated by using Euclidean and was decomposed to obtain the principal coordinates. We selected the first two principal coordinates for visualization. Based on the taxonomic annotation and relative abundance outcomes, bar-plot diagram analysis was conducted using R software (v3.6.0). The difference in the dominant bacterial communities was distinguished using Linear discriminant analysis Effect Size (LEfSe) analysis [25]. Firstly, the ANOVA test was used to detect different features with a threshold set at 0.05. Secondly, the Wilcoxon rank sum test was employed to analyze the differences among subgroups, with a threshold set at 0.05. Finally, Linear Discriminant Analysis (LDA) was employed to evaluate the influence of species with significant differences. The threshold was set at 3. The prediction of KEGG pathways functions based on the 16S rRNA data was performed by Phylogenetic Investigation of Communities by Reconstruction of Unobserved States 2 (PICRUSt2) [26].

### 2.6 Statistical Analysis

IBM SPSS Statistics software was used to analyse the demographic and clinical characteristics of the participants. Group comparisons were performed using the Chi-square test, Wilcoxon rank sum test, and Analysis of Variance (ANOVA). Spearman's correlation was used for correlation analysis. Random forest models were constructed with 10-fold cross-validation to identify potential biological markers that could distinguish subgroups. 70% of subjects were employed as a training set, and the remaining as a test set. The number of discriminating genera was decided based on CV error. The genus selection of oral microbiota for the predictive model was based on the top 5 mean decrease of accuracy. With the combination of the top 5 genera, we constructed the predictive model by the package ‘randomForest’ in R (v 3.6.0). The model’s performance was evaluated using the Area Under the Curve (AUC) to assess the discriminative ability of the model in predicting clinical outcomes. The statistical significance was considered at *p* < 0.05.

## 3 RESULTS

### 3.1 Clinical Characterization of the Study Population

Initially, 53 patients diagnosed with Crohn's disease were enrolled. After excluding 6 who were lost during follow-up, 47 CD patients completed the entire UST therapy and ultimately received an assessment of UST efficacy (Fig. **1**). Clinical response was assessed in 68.09% of the patients (n=32) and clinical remission was assessed in 42.55% of the patients (n=20). The demographic characteristics of the subjects are presented in Table **S2**.

### 3.2 The Composition of the Oral Microbiota in CD Patients and Healthy Controls (HC)

**Fig. (2):**
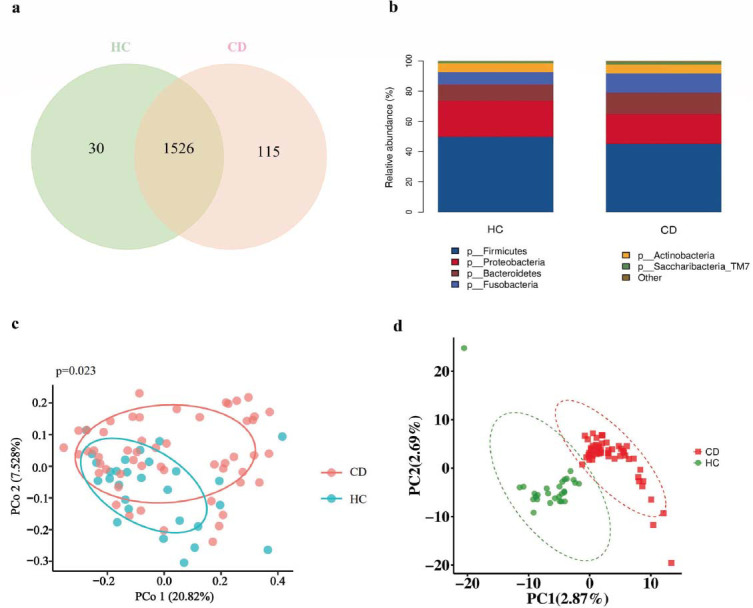
The oral microbial differences between HC and CD groups. (**a**) Venn diagram showing the uniqueness and repeatability of species composition between the two groups. (**b**) Oral microbial composition at the genus level. (**c**) Beta analysis based on PCoA of Euclidean distance (*P* = 0.010). (**d**) PLS-DA plot classifying HC and CD groups.

**Fig. (3):**
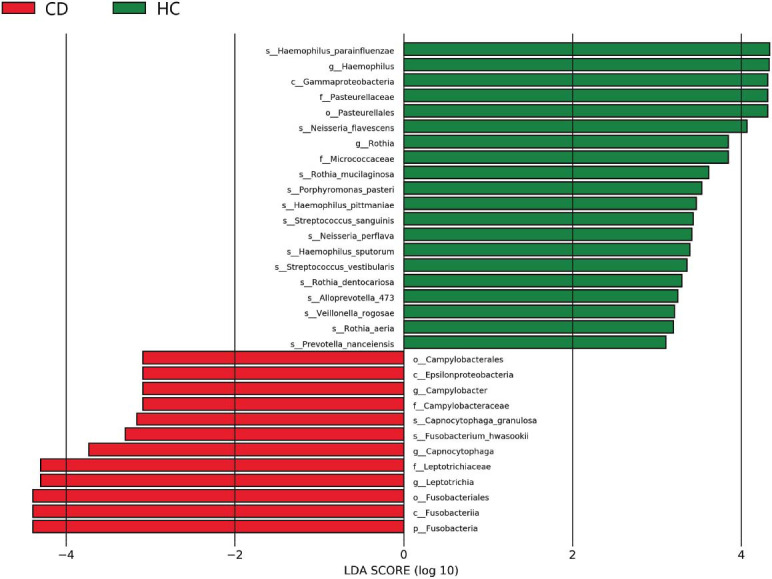
The differences in microbiota between the CD and HC groups represented by LEfSe analysis. LDA scores were set at 3 (*p*: phylum, c: class, o: order, f: family, g: genus, s: species).

We investigated differences in the oral microbiota between individuals with CD and HC. The rarefaction curves for all oral microbiota samples plateaued after an initial steep increase (Fig. **S1**). The results of 16S rRNA gene sequencing revealed that out of 1641 ASVs, 1524 were shared between the HC and CD groups, with 115 being unique to the CD group (Fig. **2a**). At the phylum level, *Firmicutes*, *Proteobacteria*, *Bacteroidetes*, *Fusobacteria*, *Actinobac- teria*, and *Saccharibacteria_TM7* were the predominant taxa in both groups (Fig. **2b**). However, no significant differences in alpha diversity were observed. Beta diversity analysis using the Euclidean distance metric revealed a significant distinction between the two groups (*P* = 0.010) (Fig. **2c**). This was further supported by Partial Least Squares Discriminant Analysis (PLS-DA), which clearly separated the two groups (Fig. **2d**). Linear discriminant analysis Effect Size (LEfSe) identified a significantly higher abundance of *Fusobacteria* in CD patients compared to HC. At the genus level, CD patients exhibited an enrichment of *Leptotrichia*, *Capnocytophaga*, and *Campylobacter*, with a diminution in *Haemophilus* and *Rothia* (Fig. **3** and Fig. **S1a**). The normalized relative abundances of discriminative bacterial taxa between CD and HC identified by LEfSe analysis are presented in the form of a heatmap (Fig. **S2**).

### 3.3 Relationship Between Oral Microbiota and Clinical Outcomes

**Fig. (4):**
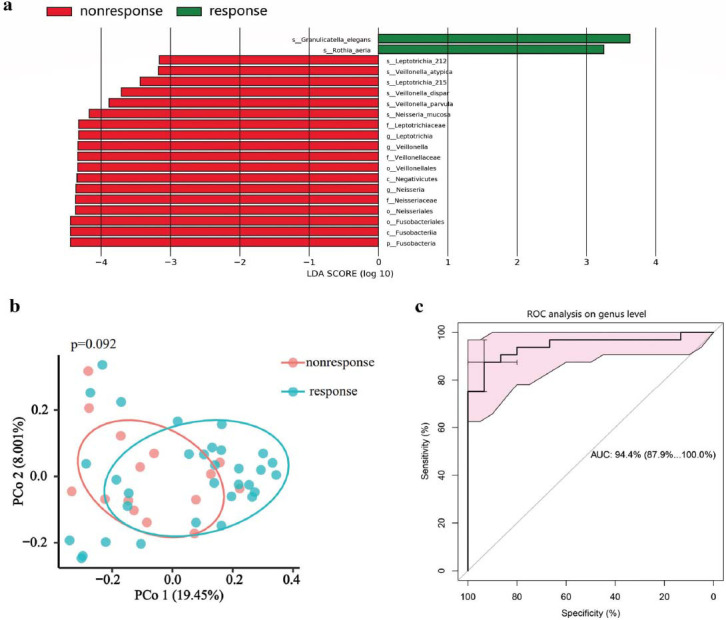
The oral microbial differences between the response group and the non-response group. (**a**) LEfSe analysis among response and non-response groups. (**b**) Beta analysis based on PCoA of Euclidean distance (*P* = 0.033). (**c**) Random forest model for classifying responders and non-responders to UST based on the top 5 genera.

**Fig. (5):**
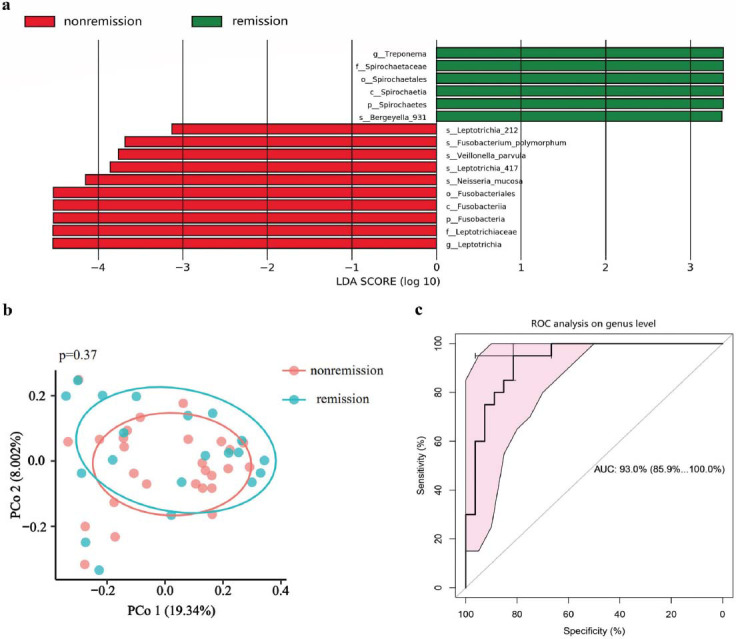
The oral microbial differences between the remission group and the non-remission group. (**a**) LEfSe analysis among remission and non-remission groups. (**b**) Beta analysis based on PCoA of Euclidean distance (*P* = 0.185). (**c**) Random forest model for classifying the remission group and non-remission group based on the top 5 genera.

Alpha diversity analysis revealed no statistically significant differences between the response and non-response groups, nor between the remission and non-remission groups. (Figs. **4** and **5**) However, beta diversity analysis using PCoA of the Euclidean distance metric showed a significant difference between the response and non-response groups (Fig. **4b**), while no such difference was observed between the remission and non-remission groups (Figs. **5a** and **5b**). The result of LEfSe analysis further demonstrated a higher relative abundance of *p_Fusobacteria*, *g_Neisseria*, *g_Veillonella*, *g_Leptotrichia* in the non-response group (Fig. **4a** and Fig. **S1b**). Noteworthily, the enrichment of *p_Fusobacteria* and *g_Leptotrichia* was observed in the non-remission group as well. In contrast, the non-remission group exhibited a reduction in *p_Spirochaetes* and *g_Treponema*, compared to the remission group (Fig. **5a** and Fig. **S1c**). The heatmap displayed the normalized relative abundances of discriminative bacterial taxa between response and non-response groups (Fig. **S3a**) and remission and non-remission groups (Fig. **S3b**), as identified by LEfSe analysis.

### 3.4 Metabolic Functional Variation Predicted by PICRUSt2

**Fig. (6):**
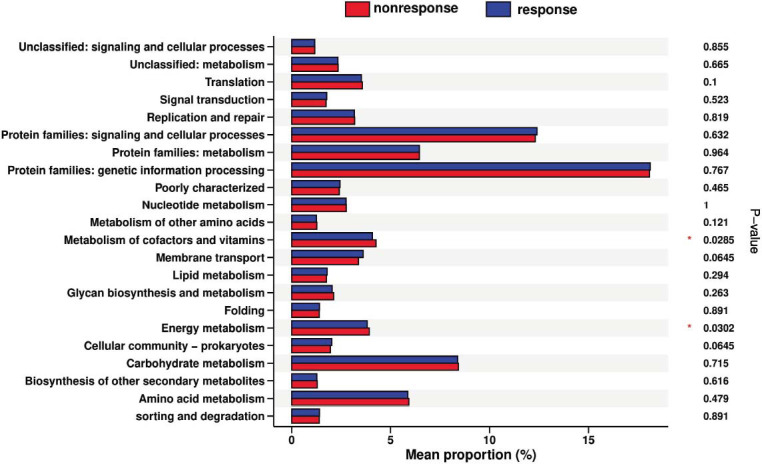
KEGG pathways by PICRUSt2 showing the oral microbial functional differences between the response group and the non-response group.

Based on the 16S rRNA sequence, we further utilized PICRUSt2 to investigate the association of oral microbiota metabolic pathways and UST clinical efficacy. The results indicated that energy metabolism (*P* = 0.030) and metabolism of cofactors and vitamins (*P* = 0.029) were enhanced in the non-response group (Fig. **6**). However, no significant functional differences were found between the remission group and the non-remission group. The PICRUSt2 analysis revealed that the non-response group had a dysfunctional microbial composition and metabolic dysfunction in the microbial community, which could be relevant to the response to UST in CD patients.

### 3.5 Predictive Model of UST Efficacy in CD Patients

We analysed the Operational Taxonomic Units (OTUs) of 47 CD patients who underwent an assessment of UST efficacy and constructed Random Forest (RF) predictive models based on the genus level of the oral microbial composition. The top five microbial markers of importance distinguishing the responders and the non-responders were *Paracoccus*, *Veillonella*, *Atopobium*, *Olsenella*, and *Ochrobactrum* (Fig. **S4a**), while the top five biomarkers of the remission and non-remission groups were *Bacteroidetes_G.5*, *Leptotrichia*, *Treponema*, *Lachnospiraceae_G.8*, and *Rhodocyclus* (Fig. **S4b**). The response group was separated from the non-response group with an Area Under the receiver operating Characteristic curve (AUC) of 0.944 (95%CI: 0.879-1.000) (Fig. **4c**). The predictive model for classifying patients into remission and non-remission groups showed an AUC value of 0.930 (95%CI: 0.859-1.000) (Fig. **5c**).

## 4 DISCUSSION

We observed differences in the oral microbiota between healthy individuals and CD patients. Corresponding with the study of Xun, *et al.* [18] on the oral microbiota composition in CD and HC groups, at the phylum level, *Firmicutes*, *Proteobacteria*, *Bacteroidetes*, *Fusobacteria*, *Actinobacteria*, and *Saccharibacteria_TM7* were predominant in the oral microbiota of CD patients. Compared to healthy controls, CD patients exhibited a lower relative abundance of *Haemophilus* and *Rothia*. Furthermore, we found that patients with CD had a higher abundance of *Fusobacteria*, *Leptotrichia*, *Capnocytophaga*, and *Campylobacter*, suggesting the potential oral microbiota disruption in this particular population.

Given that our study, along with a previous research work [18], indicated that the composition of microbiota differed between HCs and CD patients, we hypothesized that such differences may also exist among subgroups of CD patients. To personalize the treatment of CD patients, we further focused on the role of oral microbiota in the clinical efficacy of UST therapy for CD patients.

Our analysis of baseline oral microbiota in CD patients commencing UST treatment revealed a variation in oral microbiota among different clinical efficacy outcome subgroups. The PCoA analysis based on Euclidean showed different microbial compositions between the response group and the non-response group, which could be related to the immunoheterogeneity of CD. The oral microbiota of responders to UST appeared to be associated with the IL-12/23-related immune response, while the oral microbiota of non-responders did not exhibit this association. However, there was no significant difference in beta diversity between the non-remission group and the remission group. This could be attributed to the fact that some patients who were defined as responders did not meet the standard for remission and were, therefore, classified into the non-remission group. This may have weakened the relationship between oral microbial composition and clinical efficacy when the clinical outcome variable was remission and non-remission.

Accumulating studies have confirmed the role of oral microbiota in promoting intestinal inflammation [27-29]. Due to the multiple protective barriers of the gastrointestinal tract, oral pathogens typically cannot colonize in a healthy gut environment. However, in patients with IBD, diminished gastric acidity and compromised intestinal tight junctions facilitate the ectopic colonization of oral bacteria in the gut [19, 30]. As these oral pathogens disseminate through the intestine, they produce virulence factors, such as Lipopolysaccharide (LPS), Interleukin (IL)-17, IL-1β, TNF-α, and other virulence factors, disrupting the integrity of the intestinal barrier and aggravating both intestinal dysbiosis and chronic inflammation [31-33]. In an animal study performed by Atarashi, *et al.* [34], *Klebsiella* isolated from saliva was shown to colonize the gut and induce significant inflammation through Th1 cell activation. Additionally, oral bacteria can spread through the bloodstream during dental procedures, such as tooth brushing and dental extraction [35, 36]. Once in the systemic circulation, these oral pathogens stimulate dendritic cells and macrophages, consequently modulating the microenvironment of the gut microbiota through the induction of host immune responses [28, 37].

Moreover, LEfSe analysis revealed that a higher abundance of *Fusobacteria* and *Leptotrichia* was associated with the loss of response to UST in CD patients. Notably, *Fusobacteria* and *Leptotrichia* were also enriched in the non-remission group compared to the remission group. Furthermore, metabolic functional variation prediction analysis showed the energy metabolism pathway and metabolism of cofactors and vitamins pathway to be enriched in the non-response group. Taken together, our findings suggested that oral microbial composition might be related to the efficacy of UST in CD patients.

*Fusobacteria* are anaerobic, Gram-negative bacilli commonly found in the human oral cavity, comprising two families, *Leptotrichiaceae* and *Fusobacteriaceae*. The enrichment of *Fusobacteria* has been observed in the colon biopsies of patients with IBD and the fecal microbiota of children with severe ulcerative colitis [15, 38]. In addition, it has been found that a higher abundance of *Fusobacteria* is linked to postoperative disease recurrence in patients with CD [39]. Most prior studies of CD-associated *Fusobacteria* have focused on *Fusobacterium nucleatum*. *F. nucleatum* is traditionally well known as a periodontal pathogen, contributing to the structure of dental plaque biofilm [40]. It is rarely detected outside the oral cavity in healthy individuals, but has been found in the gut of people with IBD and colorectal cancer [41, 42]. In a study by Engevik, *et al.* [27], *F. nucleatum* was shown to secrete outer membrane vesicles in mice with gut dysbiosis, promoting intestinal inflammation. Another study on the colitis mice model discovered that *F. nucleatum* produces FadA, an adhesion, which enhances the pathogen's acid resistance under disease conditions, facilitating its colonization of the gut [43].

*Leptotrichia*, another genus within *Fusobacteria*, is an opportunistic pathogen found in the oral cavity, intestinal tract, and urogenital system [44]. It has been reported that *Leptotrichia* is related to septicemia in patients with mucositis, oral lesions, wounds, and abscesses [45, 46]. A recent observation has confirmed the enrichment of Leptotrichia in the intestinal environment of Ulcerative Colitis (UC) [47]. A retrospective clinical study spanning 10 years indicated *Leptotrichia* to be associated with invasive infections, particularly in immunocompromised individuals [48]. Langfeldt, *et al.* [49] found that *Leptotrichia* could induce the transcription of proinflammatory factors, including IL-1β, IL-6, IL-8, and IL-10, in epithelial cells. The enrichment of *Fusobacteria* and *Leptotrichia* has been found to be related to a reduced risk of pancreatic cancer. The researchers have assumed that it could profit from the immune response elicited by *Leptotrichia*, providing the protection against pancreatic carcinogenesis [50]. It is likely that this immune mechanism could contribute to the autoimmune response seen in patients with CD, exacerbating intestinal inflammation. Therefore, *Fusobacteria* and *Leptotrichia* could serve as potential therapeutic targets for CD. Further investigations into the pathogenesis of *Leptotrichia* and other *Fusobacteria* species in gastrointestinal diseases are warranted.

Furthermore, we developed RF models to predict the efficacy of UST in CD. The AUC for the predictive model distinguishing responders and non-responders was 0.944, while the AUC for the model predicting clinical remission was 0.930. The results suggested that the oral microbial composition showed a great ability to classify CD patients with different UST efficacy. Our study is the first to verify the association of oral microbiota and the clinical outcome of UST in CD. It may further allow the identification of CD patients with a higher likelihood of UST response based on oral microbiota. Moreover, compared to fecal microbiota and mucosal biopsy, oral microbiota samples are non-invasive and easily obtainable. If validated in larger cohorts, this classification model could be used to guide UST treatment decisions in CD patients.

Nevertheless, several limitations existed in our study. Our research work, as a single-time point research, could not establish the causality between the oral microbiota composition and the efficacy of UST in patients with CD. We recruited the family members of patients as controls. However, family members may share genetic predispositions or environmental factors with CD patients, which could result in similarities in oral microbiota composition, thereby reducing the differences between CD patients and healthy controls. Moreover, potential confounding factors, such as oral hygiene behaviour and socio-economic factors, were not accounted for in our analysis. Diet having a known influence on both oral microbiota and CD symptoms may have also affected our results. Furthermore, by focusing on the top five genera, our random forest analysis may have overlooked more subtle interactions. Given the lack of research linking oral microbiota to UST efficacy in CD, a larger randomized cohort is needed to confirm the observed variations in oral microbiota and its predictive value as a biomarker. As a pilot study, our research could benefit from an expanded cohort and multicenter trials to validate and broaden the applicability of these findings.

## CONCLUSION

In our study, we have identified differences in the oral microbiota between Healthy Controls (HC) and CD patients. Compared to HCs, *Fusobacteria* and *Leptotrichia* were more abundant in CD patients. A higher abundance of *Fusobacteria* and *Leptotrichia* was also observed in the non-response and non-remission groups, compared to patients who responded to and achieved remission with UST treatment. It is possible that *Fusobacteria* and *Leptotrichia* exacerbate intestinal inflammation in CD through host immune responses. Further exploration is needed to understand the specific mechanisms by which *Fusobacteria* and *Leptotrichia* influence the efficacy of UST in patients with CD. We also constructed predictive models for clinical response (AUC: 0.944) and clinical remission (AUC: 0.930) based on the oral microbiota. The oral microbiota might be related to the efficacy of UST in CD patients and could serve as a non-invasive prognostic biomarker for UST treatment in the future. However, validation in larger cohorts is necessary.

## Data Availability

The data used in this study will be shared upon reasonable request to the corresponding author.

## LIST OF ABBREVIATIONS

CD: Crohn’s Disease
UST: Ustekinumab
HC: Healthy Control
AUC: Area Under the Curve
MCP-1: Moattractant Protein-1
PD: Probing Depth
PCoA: Principal-coordinate Analysis
LDA: Linear Discriminant Analysis
OTUs: Operational Taxonomic Units
RF: Random Forest
HC: Healthy Controls

## STANDARDS OF REPORTING

STROBE guidelines were followed.
